# Effects of Spherical and Rod-like Gold Nanoparticles on the Reactivity of Human Peripheral Blood Leukocytes

**DOI:** 10.3390/antiox13020157

**Published:** 2024-01-26

**Authors:** Patrycja Talarska, Paulina Błaszkiewicz, Artur Kostrzewa, Przemysław Wirstlein, Michał Cegłowski, Grzegorz Nowaczyk, Alina Dudkowiak, Beniamin Oskar Grabarek, Paulina Głowacka-Stalmach, Agnieszka Szarpak, Jakub Żurawski

**Affiliations:** 1 Department of Immunobiology, Poznan University of Medical Sciences, 60-806 Poznan, Poland; akost@ump.edu.pl (A.K.); zurawski@ump.edu.pl (J.Ż.); 2 Faculty of Materials Engineering and Technical Physics, Poznan University of Technology, 60-965 Poznan, Poland; paulina.blaszkiewicz@put.poznan.pl (P.B.); alina.dudkowiak@put.poznan.pl (A.D.); 3 Division of Reproduction, Department of Obstetrics, Gynecology, and Gynecologic Oncology, Poznan University of Medical Sciences, 60-535 Poznan, Poland; abys@ump.edu.pl; 4 Faculty of Chemistry, Adam Mickiewicz University Poznań, 61-614 Poznan, Poland; michal.ceglowski@amu.edu.pl; 5 NanoBioMedical Centre, Adam Mickiewicz University Poznań, 61-614 Poznan, Poland; nowag@amu.edu.pl; 6 Collegium Medicum, WSB University, 41-300 Dabrowa Gornicza, Poland; beniamin.grabarek@wsb.edu.pl (B.O.G.); pglowacka@wsb.edu.pl (P.G.-S.); 7 Faculty of Medicine, Uczelnia Medyczna im. Marii Skłodowskiej-Curie, 00-136 Warszawa, Poland; szarpak.adr@gmail.com

**Keywords:** gold nanoparticles (GNPs), reactive oxygen species (ROS), cellular toxicity, leukocytes, caspase-1 activity, interleukin-1β (IL-1β) concentration

## Abstract

Gold nanoparticles (GNPs) are widely used in the technological and biomedical industries, which is a major driver of research on these nanoparticles. The main goal of this study was to determine the influence of GNPs (at 20, 100, and 200 μg/mL concentrations) on the reactivity of human peripheral blood leukocytes. Flow cytometry was used to evaluate the respiratory burst activity and pyroptosis in monocytes and granulocytes following incubation with GNPs for 30 and 60 min. Furthermore, the concentration of interleukin-1β (IL-1β) in human blood samples was assessed using enzyme-linked immunosorbent assay (ELISA) after their incubation with GNPs for 24 h. Under the conditions tested in the study, the GNPs did not significantly affect the production of reactive oxygen species in the granulocytes and monocytes that were not stimulated using phorbol 12-myristate 13-acetate (PMA) in comparison to the samples exposed to PMA (*p* < 0.05). Compared to the control sample, the greatest significant increase in the mean fluorescence intensity of the granulocytes occurred in the samples incubated with CGNPs = 100 and 200 µg/mL for tinc = 30 and 60 min (*p* < 0.05). From our results, we conclude that the physicochemical properties of the nanoparticles, chemical composition, and the type of nanoparticles used in the unit, along with the unit and incubation time, influence the induced toxicity.

## 1. Introduction

Nanoparticles are structures in which at least one dimension is between 1 and 100 nanometers (nm). This size range means they have large surface-to-volume ratios, which affects their properties. Nanoparticles can be obtained using a variety of methods, including chemical, physical, and biological. Common techniques include chemical deposition, sol-gel synthesis, and laser ablation [1]. Nanoparticles enter cells mainly through endocytosis, via the formation of endocytic vesicles and the release of ions from these vesicles into the cells. The use of nanoparticles raises concerns about their potential health and environmental impacts. Their small size and unique properties may lead to nanoparticles having biological interactions that are distinct compared to corresponding larger particles of the same material. Their unique physical and optical properties make them useful not only in medicine but also as biosensors or materials for the construction of optoelectronic devices, among other uses [2,3]. The most important advantages of using nanoparticles in medicine, including in the context of disease therapy, include the high efficiency of delivering drugs to diseased tissues when used in chemotherapy, the reduced side effects of treatment, as well as the possibility to develop new types of therapies (e.g., photothermal therapy) or targeted combined therapies, with which greater efficacy is achieved in the fight against cancer [2,3].

Gold nanoparticles (GNPs) are characterized by the ease of their assimilation by cells. As a result, they have found application mainly in the biomedical industry [4,5,6,7,8,9,10,11,12,13,14,15].

GNPs are mainly prepared according to the post-reduction reaction of tetrachloroauric acid (HAuCl_4_) in a liquid medium, although they can also be obtained using photochemical methods or methods belonging to the field of “green” chemistry [4,5,6,7,8,9,10,11,12,13,14,15].

They are used as innovative tools in diagnostic tests, such as in the detection of biomarkers for cardiovascular disease or cancer, as well as in drug delivery systems [4,5,6,7,8,9,10,11,12,13,14,15].

Due to their size, GNPs also experience an enhanced permeability and retention (EPR) effect. This effect, which is observed in some neoplasms, results in increased and longer-lasting drug accumulation in lesional tissues due to the leakage of the vascular system of the tumor in question [16].

However, it should be noted that numerous reports have been published on the cytotoxicity of gold nanoparticles, dependent on their shape or shell structure, and the extent of the experimentally measured parameters [17,18].

The limitations for using GNPs in therapy and diagnostics are mainly due to the toxicity of the stabilizing agent used in their synthesis and also the stability of the nanoparticles in the biological medium and their pharmacokinetics [19].

The size-dependent toxicity of gold nanoparticles against different cell types has been characterized, demonstrating that smaller particles tend to be more toxic [17,18,20,21]. In contrast, other teams of researchers have found a lack of cytotoxic effects in GNP-treated cancer cell lines [22,23,24]. The reduced size of certain GNPs is correlated with their expanded tissue distribution, increased potential for deeper penetration into specific tissues, more effective uptake of nanoparticles by co-cells, and increased toxic effects [25]. Pan et al. showed that very small GNPs (approximately 1.4 nm) cause cellular apoptosis due to oxidative stress and mitochondrial damage [26].

Using rat studies, it has been shown that gold nanoparticles can be absorbed through inhalation and ingestion [27,28]. Furthermore, research based on inhalation, administered to male subjects for 2 h during intermittent exercise, confirmed the penetration of GNPs into the lungs [29]. The ability of gold nanoparticles to pass through human skin was also confirmed in vitro using surgically resected skin [30].

With the increasing use of gold nanoparticles in biomedicine, including in therapeutics, diagnostics, imaging, and drug delivery, there is growing interest in the safety of their use [20]. Among the results of an extensive number of studies, there are also contradictory results regarding their toxicity and its dependence on their size and shape, in addition to the behavior of GNPs in biological systems [20]. Therefore, more research is needed on the effects of applied nanoparticles on the body [20]. Most pf the data in the literature are from studies conducted on cells such as HL7702 cells (human liver cell line) [31], human breast cancer cell lines SKBR-3 and MCF-7 [32], human umbilical vein endothelial cells (HUVACs) [33], murine monocytic macrophage cell line RAW264.7 [33,34], the SH-SY5Y human neuroblastoma line [35], and a human monocytic leukemia cell line (THP-1) [36]. Thus, the research presented in our paper is one of the few examples in which human peripheral blood is used as a research model. Nguyen and Falagan-Lotsch [21] also highlighted the existence of limited knowledge on the behavior of nanoparticles in biological systems [21].

In addition, our publication makes a significant contribution to the research regarding the effect of gold nanoparticles on the reactivity of human leukocytes, which may be helpful for the application of GNPs in clinical settings. In particular, the effect of two shapes of gold nanoparticles is investigated, considering three concentrations and two incubation times. To the best of our knowledge, the only other related articles in the literature describe studies on one nanoparticle type of different sizes [31], one size at three concentrations and two incubation times [32], one size at three concentrations [33], and three sizes at a single concentration [35,36].

Therefore, in this study, we aim to evaluate the respiratory burst activity of monocytes and granulocytes derived from heparinized whole blood by analyzing the fluorescence intensity in the green light spectrum of samples inoculated with GNPs at concentrations of 20, 100, and 200 μg/mL using rhodamine derivative 123 and flow cytometry. In addition, an attempt was made to evaluate pyroptosis induction in the monocytes and granulocytes using activated caspase-1 analysis of the samples incubated with gold nanoparticles in the green light spectrum using a cytometric technique. The concentration of interleukin-1β in whole blood after 24 h incubation of the blood with gold nanoparticles was also evaluated using an immunoenzymatic assay. Physicochemical characterization of the GNPs was carried out using UV–Vis spectroscopy, HRTEM microscopy, dynamic light scattering (DLS), and thermogravimetric (TG) analysis.

## 2. Materials and Methods

### 2.1. Ethical Considerations

The research described in this work was conducted in accordance with the guidelines of the 2013 Declaration of Helsinki on human experimentation. Data confidentiality and patient anonymity were maintained at all times. Patients’ identifying information was removed prior to the database analysis. Written informed consent was obtained from all patients. Consent to conduct the study was obtained from the Bioethics Committee operating at the Karol Marcinkowski Medical University in Poznań (decision number KB-33/24).

### 2.2. Reagents

The tetrachloroauric acid (HAuCl_4_·H_2_O) (99.99%) was purchased from Thermo Fisher Scientific (Thermo Fisher Scientific, Waltham, MA, USA). The sodium citrate (HOC(COONa)(CH_2_COONa)_2_·2H_2_O) (≥99.00%), cetyltrimethylammonium bromide (CTAB) (99.00%), sodium borohydride (NaBH_4_) (98.00%), silver nitrate (AgNO_3_) (99.99%), ascorbic acid (AA) (99.00%), O-(2-mercaptoethyl)-O′-methylpolyethylene glycol (PEG-SH Mw ≈ 2000) (99.99%), and saline solution were purchased from Sigma-Aldrich (Sigma-Aldrich, St Louis, MO, USA).

### 2.3. Synthesis of the Gold Nanoparticles

The spherical GNPs were obtained using a modified Turkevich method according to the manufacturer’s protocol [37,38]. A description of the synthesis process can be found in Appendix A.

The synthesis of the rod-like gold nanoparticles (GNRs) was carried out using an in situ bottom-up method [39,40,41]. A description of the complete synthesis process is presented in Appendix B.

All the tests were carried out using concentrations C_GNPs_ = 20, 100, and 200 μg/mL as calculated from the work of Scarabelli et al. [42]. A spectroscopic analytical method based on the absorbance of light at a wavelength of 400 nm, which can be measured using a UV–Vis spectrometer, is useful as a more convenient means for measuring the gold concentration. The idea is to select a wavelength at which the major contribution to the absorbance comes from the absorption related to the interband transitions in the metallic gold, which would then be used to determine the amount of gold precursor that has been reduced, regardless of the particle size and shape. An absorbance of 1.2 corresponds to [Au0] = 0.5 mM and was converted into µg/mL [42]. All the concentrations of suspended nanoparticles were prepared using isotonic sodium chloride solution (0.9%, Polpharma). A suspension buffer consisting of PEG 2000 and 0.9% saline solution was used to prepare the appropriate concentrations.

### 2.4. Physicochemical Characterization of the GNPs

All the samples were prepared under a laminar chamber. The characterization of the obtained nanostructures was carried out using UV–Vis spectroscopy (Varian Cary 4000, spectral range of 200 to 900 nm, absorbance range of 0.001 to 10) and electron microscopy (high-resolution transmission electron microscopy, HRTEM) using a JEOL ARM200F instrument (200 kV). The UV–Vis spectrometric measurements were carried out on the samples in quartz cuvettes. The HRTEM measurements were performed by depositing a colloidal dispersion of gold nanoparticles onto a copper grid covered with a thin carbon layer.

To investigate the stability of the nanoparticles over time, the UV–Vis spectra of the nanoparticles were measured before and after the study, with their size distribution determined using dynamic light scattering (DLS). A comparison of the final data with the initial results showed there were no significant differences.

The measurements were performed using the ImageJ software. Around 20 nanoparticles were considered in order to measure the average dimensions.

Thermogravimetric (TG) analysis was also performed to measure the change in mass of the studied nanoparticles resulting from the temperature increase. Thermal data were obtained using the Setaram SETSYS 1200. The samples were heated in a nitrogen stream at a heating rate of 10 °C per minute.

### 2.5. Collections of the Peripheral Blood Samples

The Regional Blood Donation and Treatment Center provided human heparinized peripheral blood samples from donors aged between 18 and 65. Prior to donation, all volunteers underwent examination by a physician and the standard blood diagnostics. The exclusion criteria were circulatory, digestive, nervous, respiratory, and urinary system diseases, infectious diseases (e.g., human deficiency virus HIV, hepatitis, syphilis), high-risk sexual behaviors, psychoactive substance use, blood diseases, psoriasis, diabetes, malignant tumors, anemia or iron deficiency, and pregnancy. Additionally, a six-month grace period was applied in cases of surgical procedures, endoscopy, tattoos, permanent makeup, piercing of the ears and other body parts, incarceration, delivery or termination of pregnancy, and stays in countries with an epidemiological situation of concern. Donors less than two weeks from the remission of disease symptoms/end of treatment, taking antibiotics, with a fever (>38 °C), inflammation symptoms, and immunodeficiencies were also excluded. In the case of female donors, the exclusion factors also included ongoing menstruation for up to three days after the end of the last period.

All the biological tests described below were started within two hours of blood collection. Whole blood was collected using a Becton Dickinson Vacutainer blood collection system with 4 mL lithium heparin (Medicallove, Gdynia, Poland, catalog number 36888). The characteristics of the blood types and the Rh system of the study participants are shown in Table 1.

### 2.6. Flow Cytometry

A CyFlow Space flow cytometer (Sysmex Partec GmbH, Goerlitz, Germany) was used for this study. Subpopulations of lymphocytes, monocytes, and granulocytes were identified among the white blood cells using FloMax software version 3.0 (Sysmex Partec GmbH, Goerlitz, Germany). The respiratory burst analysis was performed using the commercial Phagoburst test (Celonic Deutschland GmbH & Co. KG, Heidelberg, Germany, cat. no. 341058). The kit facilitated quantitative assessment of the changes in the ROS induction and respiratory burst activity in the heparinized human whole blood. The FAM FLICA™ Caspase-1 Kit caspase assay (Bio-Rad, Warsaw, Poland, cat. no. ICT097) was used to quantify pyroptosis based on the caspase-1 activity.

Cells with morphologically normal characteristics were considered for the analysis of the ROS production and caspase-1 activity in the cells. Using a DNA-staining solution derived from the Phagoburst assay in these assays allowed for the initial elimination of damaged cells or their fragments such that only normal cells were subjected to the actual cytometric analysis.

The same number of events, 10,000, was measured in each sample; these events were measured after the initial elimination of damaged cells and/or their fragments.

### 2.7. Reactive Oxygen Species Production

The induction of ROS was evaluated based on the fluorescent method, using the dihydrorhodamine 123 (DHR) included in the Phagoburst test. DHR was used to detect free radicals and reactive species such as superoxide anions (O2^•−^), hydrogen peroxide (H_2_O_2_), hydroxyl radical (OH•), peroxynitrite (ONOO^−^), nitrite (NO_2_^−^), carbonate (CO_3_^−^), and hypochlorous acid (HClO) [43,44,45].

### 2.8. Respiratory Burst

The respiratory burst of the granulocytes and monocytes was investigated by measuring the fluorescence of rhodamine 123 after DHR oxidation, upon the addition of PMA to the cells [46]. The production of H_2_O_2_ by the phagocytes was evaluated using DHR and the Phagoburst test [47].

### 2.9. Pyroptosis Analysis

Pyroptosis was evaluated using flow cytometry, as mentioned above, and the FAM-FLICA Caspase-1 Kit, which contains the FAM-VAD-FMK FLICA probe (FAM: carboxyfluorescein; VAD: derivative of valyl alanyl aspartic acid; FMK: fluoromethyl ketone; FLICA: fluorescent-labeled inhibitor of caspase) and DNA-staining fluid. The fluorescence signal was used to measure the caspase-1 activity. The tests were carried out on 22 independent blood samples, with two repetitions per sample.

### 2.10. Enzyme-Linked Immunosorbent Assay (ELISA) Tests

The concentration of interleukin-1β (IL-1β) was determined using a commercial enzyme-linked immunosorbent assay (ELISA) (human interleukin-1 beta, IL-1 beta ELISA Hölzel Diagnostika Handels GmbH, Köln, Germany, cat. no. E0563h). The samples were measured at a wavelength of 450 nm using an Epoch spectrophotometer (BioTek Instruments Inc., Winooski, VT, USA). The tests were carried out on 12 independent blood samples, with two repetitions per sample.

### 2.11. Statistical Analyses

The statistical analyses were performed using Statistica software for Windows version 13.5.0 (TIBCO Software Inc., Palo Alto, CA, USA) and PQStat version 1.8.0.476 (PQStat software, Kozieglowy, Poland). The Shapiro–Wilk test was used to assess the normality of the continuous variable distribution. The assumption of sphericity was verified using Mauchly’s test. Comparisons of related measurements were based on single-factor repeated-measures ANOVA, multivariate Wilks’ lambda for repeated measurements, MANOVA, or Friedman’s test. Tukey’s HSD, Dunnett’s tests (appropriate for the ANOVA and Wilks’ lambda multivariate test), and Dunn’s test (with Friedman’s) were used as the post hoc tests. The t-test, or the Wilcoxon pair-order test, was used in cases of two dependent groups. The results are presented as mean ± SD (standard deviation). The data were considered statistically significant different at *p* < 0.05.

## 3. Results

### 3.1. Physicochemical Characterization of the GNPs

#### 3.1.1. UV–Vis

The UV–Vis spectroscopic analysis (Figure S1. UV–Vis spectrum of spherical gold nanoparticles (A) (λmax = 526 nm; optical path = 10 mm) and rod-shaped gold nanoparticles (B) (high peak λ = 685 nm, low peak λ = 518 nm; optical path = 10 mm)) confirmed the formation of small and uniform nanoparticles, as evidenced by a narrow absorption band at λ_max_ = 526 nm.

The UV–Vis spectral image is consistent with the results in the literature. Smaller and spherical gold nanoparticles show absorption around 530 nm and are characterized by narrow peaks [48,49].

The spectrophotometric UV–Vis analysis (Figure S1. UV–Vis spectrum of spherical gold nanoparticles (A) (λmax = 526 nm; optical path = 10 mm) and rod-shaped gold nanoparticles (B) (high peak λ = 685 nm, low peak λ = 518 nm; optical path = 10 mm)) further confirmed the characteristic occurrence of two peaks, at 685 nm (high peak) and 518 nm (low peak), for the rod-like gold nanoparticles.

The UV–Vis spectral image is consistent with the reports of other authors. The rod-like gold nanoparticles show absorption of a higher peak around 680 nm and a lower peak around 530 nm [48,49].

#### 3.1.2. HRTEM

Electron microscopy (HRTEM) characterization of the obtained GNPs confirmed their spherical shape and an average size of 13.24 ± 1.80 nm (Figure S2. High-resolution transmission microscope image of spherical gold nanoparticles (A), with an average diameter of 13.24 ± 1.80 nm, and rod-shaped gold nanoparticles (B), with an average diameter of 31.92 ± 4.47 × 11.85 ± 3.13 nm).

Furthermore, HRTEM characterization of the obtained GNRs confirmed their rod shape with an average size of 31.92 ± 4.47 × 11.85 ± 3.13 nm (Figure S2. High-resolution transmission microscope image of spherical gold nanoparticles (A), with an average diameter of 13.24 ± 1.80 nm, and rod-shaped gold nanoparticles (B), with an average diameter of 31.92 ± 4.47 × 11.85 ± 3.13 nm).

#### 3.1.3. Thermogravimetric Analysis

The results of the thermogravimetric analysis of the GNPs and GNRs are presented in Figure S3.

Both materials show similar behavior, presenting two major decomposition steps. The first, starting at ca. 50 °C and ending around 140 °C, is presumably associated with complete solvent removal, as only minor mass loss is observed. This mass loss is equal to ca. 6% for the GNPs and ca. 4% for the GNRs. A similar observation was reported by Natarajan et al. [50], who established that the approximately 10% mass loss occurring in the 100–200 °C range for peptide-functionalized GNPs is connected with water removal from the material surface [50].

The second step, starting at ca. 200 °C and ending at ca. 650 °C, represents the complete thermal decomposition of all the organic material present in the sample. A parallel behavior was noted for GNPs functionalized with linear thiols, where complete thiol decomposition and no further changes in the sample weight were observed for temperatures above 500 °C [51]. The total mass loss in the second step is equal to 11% for the GNPs and 9% for the GNRs. These results suggest that GNPs contain more thiol functionalities, which is presumably related to their smaller size, which results in a high surface availability. This is in accordance with the expectations that smaller nanoparticles possess a higher surface-area-to-volume ratio, with more thiol functionalities present per mass unit [52]. The fact that thermogravimetric analysis can be used for the quantification of the organic compounds present on GNP surfaces was demonstrated by Bajaj et al. [53], who employed thermogravimetric analysis to quantify the citrate ions on GNPs prepared using various synthetic procedures [53].

In the experiments, the initial masses of GNP and GNR were 10.13 mg and 10.72 mg, respectively. The temperature ramp rate was maintained at 10 °C/min, without the application of any dwelling time. The nitrogen flow rate was set at 65 cm^3^/min. For both samples, two peaks were observed in the first derivative of the temperature profile: the peaks for the GNPs occurred at 78 °C and 211 °C, while those for the GNRs were at 80 °C and 213 °C.

### 3.2. Flow Cytometry

#### 3.2.1. ROS Production

**Figure 1:**
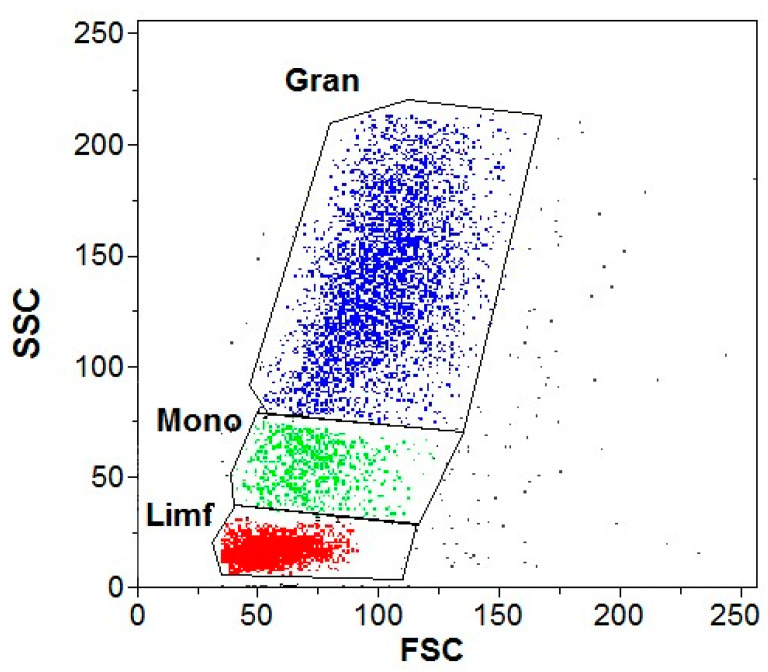
Example cytogram demonstrating gate-isolated leukocyte populations in a cytometric image without phorbol-12-myristate-13-acetate (PMA) stimulation. Gran—granulocytes; Mono—monocytes; Limf—lymphocytes.

DHR was used for all the experiments. The differentiating element was the addition of GNPs to the test samples and buffer to the control samples. An example cytogram of the gate-isolated leukocyte populations in a cytometric image is shown in Figure 1.

**Figure 2:**
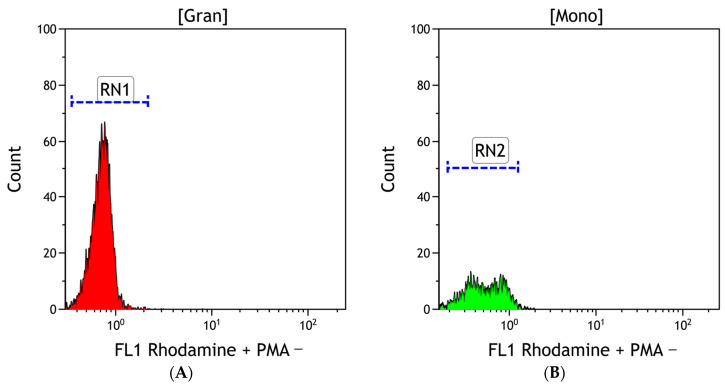
Histogram showing the fluorescence intensity value of granulocytes (**A**) and monocytes (**B**) and demonstrating rhodamine fluorescence 123 without phorbol-12-myristate-13-acetate (PMA) stimulation, generated using the Kaluza Analysis 2.2.1 software (Beckman Coulter, Boston, MA, USA).

The percentage of cells producing ROS was analyzed, with their mean fluorescence intensity measured (Figure 2). The tests were carried out on 22 independent blood samples, with two repetitions per sample.

**Figure 3:**
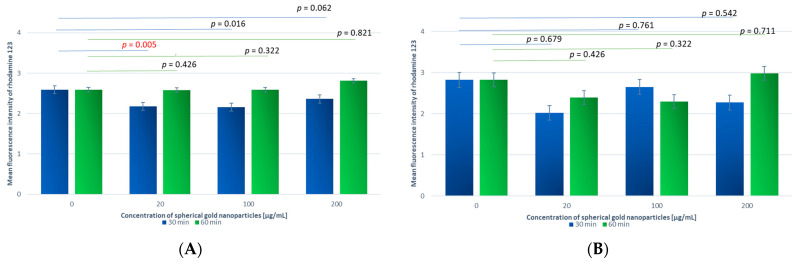

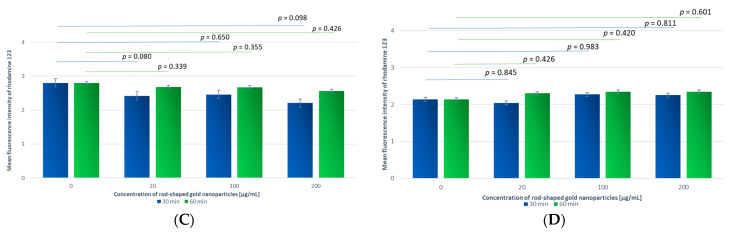
Reactive oxygen species production by granulocytes (**A**) and monocytes (**B**) incubated with spherical gold nanoparticles and by granulocytes (**C**) and monocytes (**D**) incubated with rod-shaped gold nanoparticles, evaluated based on the rhodamine 123 fluorescence intensity measured using flow cytometry. Horizontal lines indicate differences between incubation times related to GNP and GNR concentrations, where blue lines are for 30 min incubation time and green lines are for 60 min incubation time.

The study was conducted on 22 independent blood samples, with two replicates per sample. We found very small differences in the values of the mean rhodamine fluorescence intensity of both the granulocytes and monocytes between all samples incubated with gold nanoparticles compared to the control sample. For the spherical gold nanoparticles (Figure 3A,B), the result was observed to be directly and proportionally dependent on concentration, i.e., the highest values of the mean fluorescence intensity were detected for the highest concentration. No such dependence was observed for the rod-like gold nanoparticles (Figure 3C,D).

The distribution of the sample results is shown in the dot plot (Figure S4. Reactive oxygen species production by granulocytes (A) and monocytes (B) incubated with spherical gold nanoparticles and by granulocytes (C) and monocytes (D) incubated with rod-shaped gold nanoparticles, evaluated according to the rhodamine 123 fluorescence intensity measured using flow cytometry).

Of the analyzed samples, our results showed statistically significant differences for the samples incubated with GNPs for 30 min at concentrations of 20 and 200 µg/mL compared to the control sample. Of the samples incubated with GNRs, the results showed no statistically significant differences compared to the control sample.

##### A Comparison of Samples Incubated with GNPs and GNRs for Different Time Periods

The value of the mean rhodamine fluorescence intensity was compared for granulocytes incubated with spherical gold nanoparticles at C_GNP_ = 20 µg/mL for t_inc_ = 30 min and 60 min. The results indicate a significant difference between these samples (*p* = 0.005). The mean fluorescence intensity was higher for t_inc_ = 60 min (2.58 ± 1.91) than t_inc_ = 30 min (2.18 ± 1.42).

The mean fluorescence intensity was measured for rhodamine-stained granulocytes incubated with GNRs at C_GNR_ = 200 µg/mL for two different time periods (30 and 60 min). A statistically significant difference was found between the analyzed samples (*p* = 0.037). A higher value of the mean fluorescence intensity was recorded for t_inc_ = 60 min (2.56 ± 1.68) than t_inc_ = 30 min (2.21 ± 1.00). No statistically significant difference was found for the other samples.

##### A Comparison of Samples Incubated with GNPs or GNRs at Different Concentrations

An evaluation of the difference in the mean fluorescence intensity of the samples depending on the concentration of nanoparticles was carried out at t_inc_ = 30 min and 60 min. The results confirmed the statistical significance of the difference (*p* < 0.001) only for the GNP samples incubated for t_inc_ = 30 min. Using Dunn’s post hoc test, it was confirmed that the nanoparticle concentration dependence was determined by the difference in the mean fluorescence intensity between C_GNP_ = 20 µg/mL vs. C_GNP_ = 200 µg/mL and C_GNP_ = 100 µg/mL vs. C_GNP_ = 200 µg/mL (*p* < 0.05 for both comparisons). In both cases, a higher value was observed at C_GNP_ = 200 µg/mL. No statistically significant difference was found for the other comparisons.

##### A Comparison of Samples Incubated with Different Shapes of Gold Nanoparticles

The values of the mean fluorescence intensity of the pairs were compared between the rhodamine-containing blood samples incubated for t_inc_ = 30 min with GNPs or GNRs at various concentrations. At C_GNPs_ = 100 µg/mL, the difference in the mean fluorescence intensity between the GNP and GNR samples proved to be statistically significant. A higher value was recorded for GNRs, for which it was 2.46 ± 1.75 compared to 2.16 ± 1.31 for GNPs. In contrast, for analogous pairs of samples at C_GNPs_ = 20 and 200 µg/mL, the differences were not statistically significant (*p* = 0.194 and *p* = 0.178, respectively).

For the rhodamine blood samples incubated with GNPs or GNRs for t_inc_ = 60 min at different concentrations (C_GNPs_ = 20, 100, 200 µg/mL), there was no significant difference between the types of nanoparticles at any of the concentrations used.

#### 3.2.2. PMA-Addition-Stimulated Production of Reactive Oxygen Species

**Figure 4:**
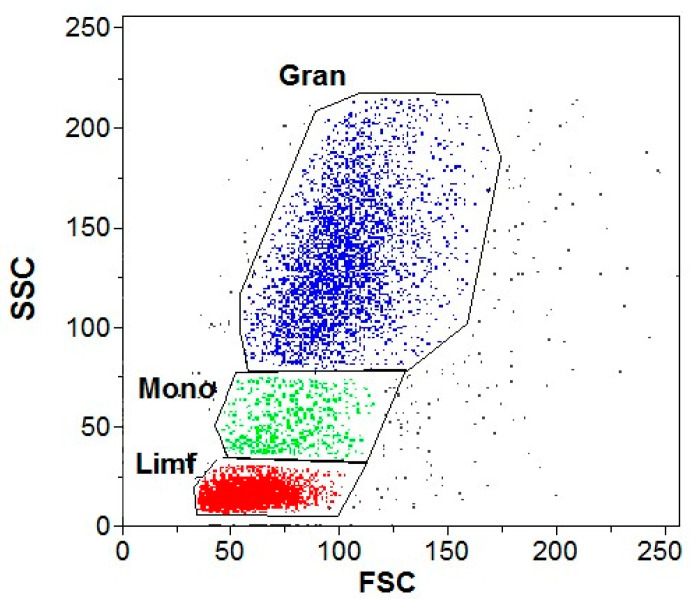
Example cytogram showing gate-isolated populations of leukocytes stimulated by PMA in a cytometric image. Gran—granulocytes; Mono—monocytes; Limf—lymphocytes.

DHR and PMA were used for all the samples in which a respiratory burst was examined. The differentiating element was the addition of GNPs to the test samples and buffer to the control samples. An exemplary cytogram showing gate-isolated populations of leukocytes stimulated by PMA in a cytometric image is shown in Figure 4.

**Figure 5:**
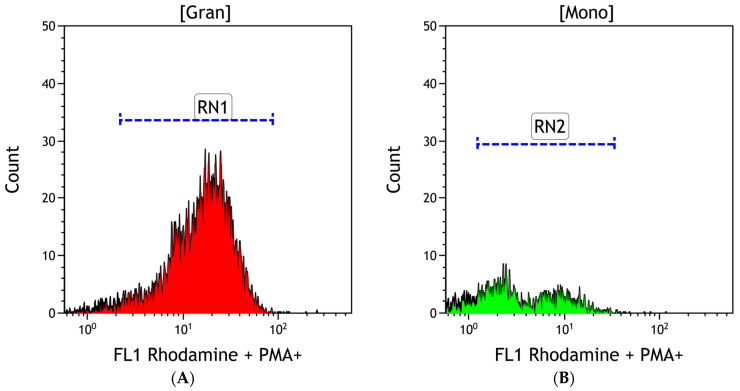
Example histogram showing the fluorescence intensity value of granulocytes (**A**) and monocytes (**B**) exhibiting rhodamine 123 fluorescence stimulated by phorbol-12-myristate-13-acetate (PMA), generated using the Kaluza Analysis 2.2.1 software (Beckman Coulter, Boston, MA, USA).

The percentage of cells exhibiting signs of respiratory burst and the mean fluorescence intensity of the DHR-labeled cells were analyzed (Figure 5).

The tests were carried out on 22 independent blood samples, with two repetitions per sample.

The percentage values of granulocytes participating in the respiratory burst correspond to those described by the manufacturer of the Phagoburst assay and remained at an average level of 94.13 ± 4.25%. In the case of the monocytes, the percentage values remained at an average of 80.75 ± 5.44%. Statistical analysis of the percentage values showed no significant differences between any samples.

**Figure 6:**
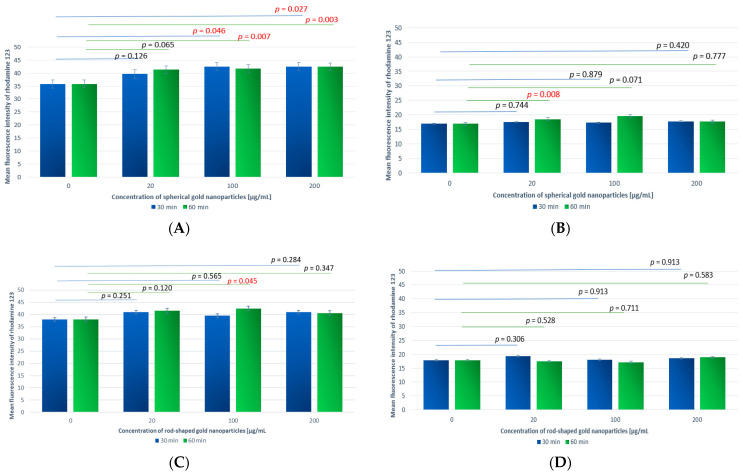
Reactive oxygen species production by granulocytes (**A**) and monocytes (**B**) incubated with spherical gold nanoparticles and by granulocytes (**C**) and monocytes (**D**) incubated with rod-shaped gold nanoparticles after stimulation with PMA, evaluated based on the rhodamine 123 fluorescence intensity measured using flow cytometry. Horizontal lines indicate differences between incubation times related to GNP and GNR concentrations, where blue lines are for 30 min incubation time and green lines are for 60 min incubation time.

A large increase in the mean rhodamine fluorescence intensity was observed after the addition of PMA, mainly in the granulocytes. There was also an increase in respiratory burst for all the samples incubated with the GNPs compared to the control sample. The greatest significant increase in the mean fluorescence intensity of the granulocytes compared to the control sample occurred in the samples incubated with C_GNPs_ = 100 and 200 µg/mL for t_inc_ = 30 and 60 min (Figure 6A).

An increase in the mean fluorescence intensity of the granulocytes was also observed in the samples incubated with the GNRs. The greatest significant increase compared to the control sample occurred at C_GNR_ = 100 µg/mL and t_inc_ = 60 min (Figure 6C).

Among the monocytes, a smaller increase in the mean fluorescence intensity was observed in the samples incubated with the GNPs compared to the control samples. The highest difference occurred for C_GNP_ = 100 µg/mL and t_inc_ = 60 min. However, the difference demonstrated no statistical significance (Figure 6B).

Similarly, little change in the fluorescence intensity was observed in the samples incubated with GNRs in the monocytes. No significant differences were noted (Figure 6D).

The distribution of the sample results is shown in a scatter plot (Figure S5. Reactive oxygen species production by granulocytes (A) and monocytes (B) incubated with spherical gold nanoparticles and by granulocytes (C) and monocytes (D) incubated with rod-shaped gold nanoparticles after stimulation with PMA, evaluated based on the rhodamine 123 fluorescence intensity measured using flow cytometry).

##### Comparison of Samples Incubated for 30 min with GNPs at Different Concentrations

The mean rhodamine fluorescence intensity of the PMA-treated samples incubated for t_inc_ = 30 min with GNPs was 39.58 ± 17.92 at C_GNP_ = 20 µg/mL and 42.47 ± 19.29 at C_GNP_ = 200 µg/mL. The highest value of 42.55 ± 19.61 was recorded at C_GNP_ = 100 µg/mL. The correlation between the mean fluorescence intensity of the samples and the nanoparticle concentrations was evaluated for the same incubation time of t_inc_ = 30 min, yielding certain statistically significant differences (*p* = 0.033). However, Tukey’s HSD post hoc test showed no indication of a pair between which the difference was statistically significant. However, in the case of the samples with a GNP concentration between 20 µg/mL and 100 µg/mL, the significance level was relatively close to the assumed threshold (*p* = 0.054). This result indicates that, in the overall comparison, this pair most likely contributes the most to the statistical significance of the results regarding the dependence of the mean fluorescence intensity value on the nanoparticle concentration (according to Tukey’s HSD post hoc test, *p* < 0.05).

A comparison between the other granulocyte-containing samples showed there was no statistically significant difference due to the type of gold nanoparticle used (GNP or GNR), concentration (C_GNPs_ = 20, 100, 200 µg/mL), or incubation time (t_inc_ = 30 or 60 min).

##### Comparison of Samples Incubated with GNRs at the Same Concentration and Different Incubation Times

Analysis of the mean rhodamine fluorescence intensity of the monocytes treated with PMA and incubated with GNRs at C_GNR_ = 20 µg/mL for t_inc_ = 30 and 60 min showed a significant difference between these samples (*p* = 0.014). The mean fluorescence value of the intensity was higher for t_inc_ = 30 min (19.29 ± 3.63) than t_inc_ = 60 min (17.39 ± 4.76).

A comparison between the other monocyte-containing samples showed there was no statistically significant difference due to the type of gold nanoparticle used (GNP or GNR), concentration (C_GNPs_ = 20, 100, 200 µg/mL), or incubation time (t_inc_ = 30 or 60 min).

### 3.3. Pyroptosis

FAM-VAD-FMK FLICA and the DNA-staining fluid were used in each of the trials. The differentiating element was the addition of GNPs to the test samples and buffer to the control samples.

**Figure 7:**
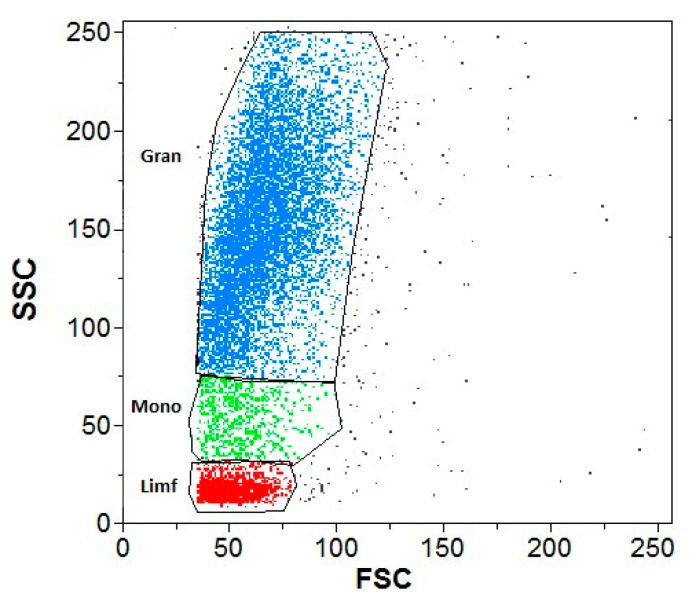
Example cytogram showing gate-isolated populations of leukocytes in a cytometric image. Gran—granulocytes; Mono—monocytes; Limf—lymphocytes.

**Figure 8:**
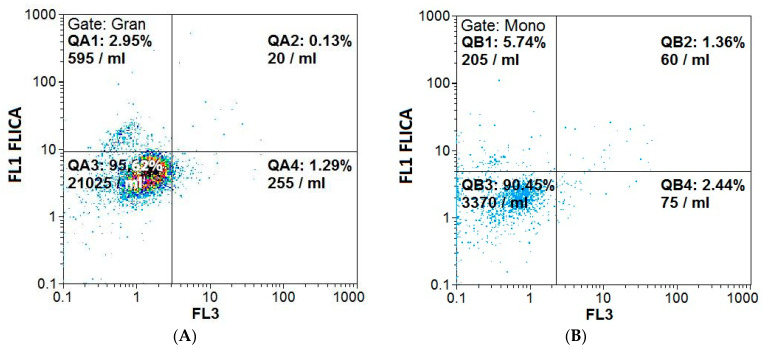
Example cytogram showing the percentage distribution of granulocytes (**A**) and monocytes (**B**) based on FAM-VAD-FMK FLICA probe fluorescence induced according to the activation of pro-inflammatory caspase-1. QA1 and QA2—cells exhibiting pyroptosis; QA3—normal cells; QA4—damaged cells or their fragments.

An example cytogram showing gate-isolated populations of leukocytes in a cytometric image is presented in Figure 7. Cytograms showing the percentage distribution of the granulocytes and monocytes are presented in Figure 8.

The differences between the percentages of normal cells and those exhibiting pyroptosis were statistically significant. In both groups of granulocytes, a significantly higher rate of normal cells was observed (mean 96.38% ± 1.33% for GNPs and 96.27% ± 1.45 for GNRs) compared to pyroptotic cells (mean 3.64 ± 1.35 and 4.18 ± 1.26, respectively) (*p* < 0.0001). Similarly, in both groups of monocytes, a significantly higher rate of normal cells was observed (mean 93.42% ± 2.81 for GNPs and 93.39% ± 2.57 for GNRs) compared to pyroptotic cells (mean 6.59 ± 2.82 and 7.05 ± 2.40, respectively) (*p* < 0.0001). For further analysis, only the cells exhibiting pyroptosis were selected.

**Figure 9:**
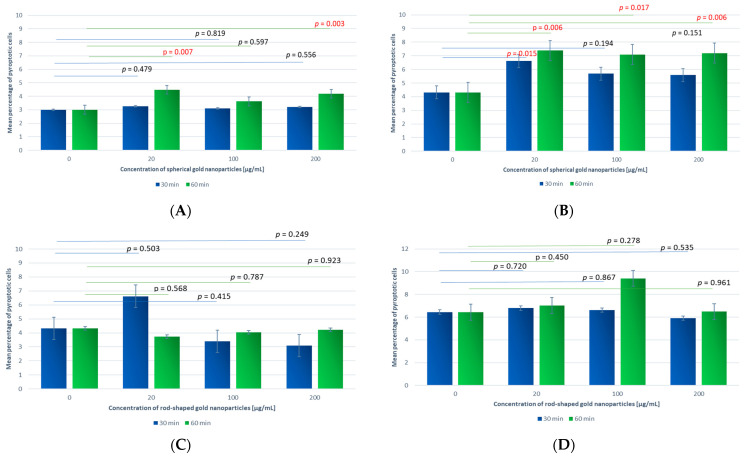
Caspase-1 activity of granulocytes (**A**) and monocytes (**B**) incubated with spherical gold nanoparticles and of granulocytes (**C**) and monocytes (**D**) incubated with rod-shaped gold nanoparticles, evaluated according to the percentage of pyroptotic cells measured using flow cytometry. Horizontal lines indicate differences between incubation times related to GNP and GNR concentrations, where blue lines are for 30 min incubation time and green lines are for 60 min incubation time.

In all the granulocyte samples incubated with GNPs, there was a slight increase in the percentage of cells exhibiting pyroptosis compared to the control sample. Statistically significant differences were observed for the samples incubated for t_inc_ = 60 min with GNPs at C_GNPs_ = 20 and 200 µg/mL compared to the control (Figure 9A).

For the granulocyte samples incubated with GNRs, there was little difference in the percentage of cells exhibiting pyroptosis, except for an increase for C_GNR_ = 20 µg/mL and t_inc_ = 30 min. No statistically significant changes were observed in the granulocyte samples compared to the control (Figure 9C).

An increase in the number of cells exhibiting pyroptosis was observed in the monocytes, indicating increased caspase-1 production compared to the control sample. The greatest increase occurred in the samples incubated with GNPs for t_inc_ = 60 min, which was statistically significant for all concentrations—20, 100, and 200 µg/mL. Significant differences were demonstrated for samples containing GNPs at C_GNP_ = 20 µg/mL and t_inc_ = 30 min (Figure 9B).

For the monocytes, a slight increase in the percentage of cells exhibiting pyroptosis was observed, especially for C_GNR_ = 100 µg/mL and t_inc_ = 60 min. In turn, a slight decrease in the percentage of cells exhibiting pyroptosis was observed for the highest concentration. There were no statistically significant differences in the comparison of the monocyte samples with the control (Figure 9D).

The distribution of the sample results is shown in a scatter plot (caspase-1 activity of granulocytes (A) and monocytes (B) incubated with spherical gold nanoparticles and of granulocytes (C) and monocytes (D) incubated with rod-shaped gold nanoparticles, evaluated according to the percentage of pyroptotic cells measured using flow cytometry).

#### Comparison between Different GNP Shapes

The samples incubated with gold nanoparticles were also compared considering the type of gold nanoparticle, GNP or GNR, as the variable for different concentrations and incubation times. There were no statistically significant differences between any of the granulocyte samples analyzed.

Furthermore, analysis of the granulocyte samples at the same concentration but for different incubation times showed the was a significant difference between the percentage of cells exhibiting pyroptosis in the samples at C_GNP_ = 20 µg/mL and t_inc_ = 30 or 60 min (*p* = 0.035). The value of the analyzed index was higher for t_inc_ = 60 min (4.48 ± 2.04) than t_inc_ = 30 min (3.26 ± 1.10). A significant difference was also observed for C_GNP_ = 200 µg/mL (*p* = 0.002). The value for the index of pyroptotic cells was higher for t_inc_ = 60 min (4.19 ± 1.44) than t_inc_ = 30 min (3.20 ± 1.01).

Moreover, a statistical difference between the samples was demonstrated for C_GNR_ = 200 µg/mL (*p* = 0.030). The value of the analyzed index was higher for t_inc_ = 60 min (4.21 ± 1.66) than t_inc_ = 30 min (3.09 ± 1.09). For the remaining comparisons, no statistically significant difference was demonstrated.

For the monocytes, there was no statistically significant difference between cells exhibiting pyroptosis in terms of the type of gold nanoparticle used (GNP or GNR), concentration (C = 20, 100, 200 µg/mL), or incubation time (t_inc_ = 30 or 60 min).

### 3.4. ELISA Results on IL-1β Concentration

Further, we examined the concentration of IL-1β in the whole blood samples after incubation with GNPs and GNRs for 24 h at C = 20, 100, and 200 μg/mL, with buffer serving as the control. The IL-1β concentration was determined based on the absorbance in comparison to the standard curve.

**Figure 10:**
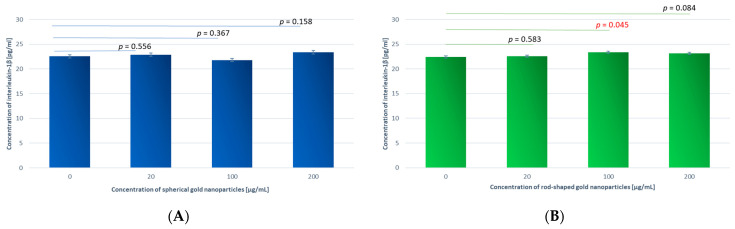
The concentration of interleukin-1β in blood samples after incubation with spherical gold nanoparticles (**A**) and rod-shaped gold nanoparticles (**B**) for 24 h measured using ELISA test. Horizontal lines indicate differences in interleukin-1β levels related to GNP and GNR concentrations.

Similar to the small differences in caspase-1 production, there was also little change in the interleukin-1β levels compared to the control sample upon 24 h incubation with the gold nanoparticles. There were also no statistically significant differences between the samples incubated with GNPs and GNRs compared to the control, except for the sample incubated with GNRs at C_GNR_ = 100 µg/mL (*p* = 0.045) (Figure 10).

The distribution of the sample results is shown in a scatter plot (the concentration of interleukin-1β in the blood samples after incubation with spherical gold nanoparticles (A) and rod-shaped gold nanoparticles (B) for 24 h measured using an ELISA test).

#### 3.4.1. Comparison of Samples Incubated with GNPs at Different Concentrations

The difference between the blood samples after incubation with GNPs at different concentrations proved to be statistically significant (*p* = 0.017), with the highest levels of interleukin-1β concentrations occurring at C_GNP_ = 200 µg/mL. In turn, no statistically significant differences were shown for the samples incubated with GNRs.

#### 3.4.2. Comparison within Samples Incubated with Different Types of Gold Nanoparticles, GNPs or GNRs, at Different Concentrations

The mean concentration of interleukin-1β was 21.79 ± 1.57 pg/mL for the GNPs and 23.36 ± 1.57 pg/mL for the GNRs. The difference between the analyzed samples at C_GNPs_ = 100 µg/mL proved to be significant (*p* = 0.006). Higher concentrations of interleukin-1β were recorded for the GNR samples. For C_GNPs_ = 20 and 200 µg/mL, the difference between the analyzed samples was insignificant.

## 4. Discussion

Coating nanoparticles with polymeric materials slows the dissolution of ions from their surface and affects their cell interaction properties [33,54,55,56].

Polyethylene glycol (PEG) does not present substantial immunogenicity, making it possible to use PEG–protein conjugates as drugs. Nanoparticles can be coated with PEG alone or PEG in combination with other molecules such as biotin, peptides, or oligonucleotides to help the GNPs better penetrate the target cells [57]. Due to their ability to bind to cell membranes, functionalized GNPs can serve as highly effective drug carriers [54].

Such GNPs are also used as nanoparticle carriers due to their biocompatibility, achieved using nanoparticle stabilization, and biosafety [35,58,59,60].

PEG-stabilized spherical and rod-like gold nanoparticles were used in our study. Several studies have found that the functionalization of GNPs’ surfaces with polyethylene glycol reduces oxidative stress. This is related to some of their properties, such as higher biocompatibility and a longer circulation time in the blood, improving the half-life of the nanobiomaterials [23,33,56,58,61,62,63,64]. Among others, it was demonstrated in Orlando et al.’s study on human umbilical vein endothelial cells (HUVACs) and the murine monocytic macrophage cell line RAW264.7 that PEGylation results in the enhanced biocompatibility of GNPs [33]. PEG also increases the half-life of the GNPs in circulation, allowing the uptake and removal of the nanoparticles by the reticuloendothelial system [20,23]. In addition, Oh and Park [65] suggest that a PEG coating may mitigate interactions between the nanoparticles and internal proteins, thereby facilitating exocytosis [65].

Dihydrorhodamine 123 was used to detect free radicals and reactive species such as O_2_^−^ and H_2_O_2_, OH−, ONOO−, NO_2_^−^, CO_3_^−^, and HOCl. This non-fluorescent dye can easily penetrate cell membranes due to its lipophilic character. Upon contact with free radicals, it oxidizes into the highly fluorescent (λem = 529 nm) cationic rhodamine 123, which is retained within the cell and accumulates in the mitochondria [43,44,45].

Oxidants and free radicals on the particle surface can directly produce reactive oxygen species. In addition, the adsorption of surrounding particulates, such as ozone and nitric oxide, onto the surface of the nanoparticles affects the induction of oxidative stress [2,66]. At low nanoparticle concentrations, cells, through their antioxidant activity, are able to defend themselves against oxidative stress and restore the redox balance. In contrast, this is not possible at higher concentrations, leading to cell cytotoxicity and the induction of inflammation [2,67]. An increase in the cytotoxicity of gold nanoparticles associated with the production of reactive oxygen species has also been observed [68,69,70,71].

Zhang et al. [35] also used PEG-functionalized nanoparticles in their study. The study was conducted on cells of the SH-SY5Y human neuroblastoma line, incubated with 4.5, 13, and 30 nm gold nanoparticles at a concentration of 25 µg/mL for 24 h. It was shown that the 4.5 nm gold nanoparticles resulted in greater toxicity than the nanoparticles of other sizes. They were also observed to have a six-fold higher level of ROS generation compared to the controls. Furthermore, mitochondrial damage in cells incubated with gold nanoparticles was confirmed according to increased DCFH-DA green fluorescence [35]. In turn, 24 h incubation of macrophages from the RAW264.7 cell line with citrate-coated 5 and 50 nm GNPs demonstrated no increase in ROS production [34].

In our study, in contrast to some literature reports, no significant increase in fluorescence dye or increased ROS production in the samples incubated with gold nanoparticles was observed [35,72]. Otherwise, our results were in line with other work where incubation with gold nanoparticles of small dimensions has resulted in altered ROS production [34,73,74].

The respiratory burst activity when phorbol-12-myristate-13-acetate was added to the cells was investigated via the measurement of rhodamine fluorescence [46].

PMA was used to activate the granulocytes and monocytes, stimulating ROS production through the direct activation of protein kinase C (PKC) and the nicotinamide adenine dinucleotide phosphate (NADPH) oxidase complex system responsible for oxidative burst in the neutrophils [75,76]. PMA is also a trigger for the formation of neutrophil extracellular traps (NETs), which can be measured by detecting extracellular DNA using DNA-staining fluid [76].

Activated cells can catalyze the NADPH-dependent reduction of O_2_ with the generation of reactive oxygen species, such as O_2_^•−^, H_2_O_2_, and OH^−^, as well as HClO. This process is known as respiratory (oxidative) burst [77,78].

The stimulation of cells with PMA resulted in the increased production of reactive oxygen species by the granulocytes and monocytes and therefore the higher fluorescence of the fluorescent dye. This effect obtained during our study is also supported by the results of numerous publications and is consistent with the manufacturer’s guidelines for the Phagoburst assay [46,77,79,80].

In our study, when a respiratory burst stimulator (PMA) was added, an increase in the production of reactive oxygen species was observed in all samples, which is consistent with the data in the literature [33,56,81], with the exception of not observing an inhibition of respiratory burst due to the gold nanoparticles [33,56,81].

In a previous study, human umbilical vein endothelial cells (HUVECs) and a mouse monocytic macrophage cell line (RAW264.7) were incubated for 6 h with 8.9 nm PEG-coated GNPs at CGNPs = 20, 50, and 100 µg/mL [33]. ROS production was observed only after the treatment of the HUVECs with PMA at all GNP concentrations. GNP treatment of the RAW264.7 macrophage line did not induce an increase in ROS production [33].

Furthermore, in a study on human neutrophil granulocytes incubated for 2 h with 20 nm GNPs, it was found that PMA-induced ROS production was inhibited by the GNPs [56].

In another study, Zhu et al. used cells from the RAW264.7 cell line incubated for 5 h with citrate-coated 5 nm GNPs at a concentration of 1 mg/mL, which showed lower DCF fluorescence compared to the sample without nanoparticles. After PMA stimulation, there was a significant increase in fluorescence for both samples. This was due to the formation of hydrogen peroxide as a result of respiratory burst. Gold nanoparticles can catalyze the formation of hydrogen peroxide under physiological conditions, thus exhibiting antioxidant properties. Studies on the RAW264.7 cell line confirmed the possibility of preventing the development of DCF fluorescence by incubating cells with GNPs. This indicates that nanoparticles can suppress the respiratory burst stimulated by PMA [81].

Oxidative stress can disrupt the lysosomes and results in protease release into the cytosol, leading to the proteolysis of cytosolic proteins and eventually resulting in cell death. This damage can lead to the leakage of lysosomal enzymes into the cytosol. When lysosomal proteases enter the cytosol, they can begin to digest cytosolic proteins, which leads to proteolysis, which, in turn, leads to the disruption of cellular processes and ultimately cell death. Thus, maintaining the redox balance in cells to prevent excessive oxidative stress is important to maintain cellular homeostasis [82,83].

Caspase-1 is the best described inflammatory caspase, inducing the maturation of IL-1β and IL-18 and pyroptotic cell death [83,84]. Pyroptosis leads to the release of cytosolic content through the formation of pores in the cell membrane, thereby enhancing the inflammatory process. Activation of the NLRP3 inflammasome complex mediates the activation of inflammatory caspase-1, crucial to the secretion of mature IL-1β and the formation of pores in the cell membrane [85]. The reactive oxygen species produced by the mitochondria also activate the NLRP3 inflammasome [86].

The results of a literature review are consistent with the results of our own study [36,62,87]. Sumbayev et al. [36] incubated a human monocytic leukemia cell line (THP-1) for 4 h with 5, 15, and 35 nm gold nanoparticles at a concentration of 20 µM. An increase in the amount of interleukin-1β on the cell surface in the presence of GNPs was demonstrated, combined with the attenuation of the IL-1β-induced inflammatory responses. This suggests that IL-1β particles aggregate around the GNPs, reducing the number of available IL-1β molecules that can interact with the cellular interleukin receptor. As a result, they significantly inhibit the biological activity of IL-1β. GNPs do not activate the inflammasome pathway in THP-1 cells and can reduce the inflammatory process by selectively targeting the IL-1β-dependent pathway. Moreover, in mouse studies based on 4-week injections of gold nanoparticles, GNPs were confirmed to reduce the IL-1β-induced response in vivo [36]. In turn, Zeng et al. assessed the effect of GNPs coated with PEG or citric acid on the function of blood cells and their distribution in individual blood cells. These authors demonstrated that PEGylation may result in their ability to escape the immune system. Regardless of the substance used to cover their surface, the use of GNPs results in an increase in platelet concentration; however, further research is required to substantiate this observation [62]. In Anik et al., it was demonstrated that the association of GNPs with an antigen present on the cell surface causes an immunizing effect, resulting in the increased secretion of cytokines, including IL-1, that induce B lymphocytes [87].

In our study, after the addition of PMA, GNPs stimulated reactive oxygen species production, caspase-1 activity, and increases in the concentration of interleukin-1β. Considering this aspect, the obtained results show little variation.

Furthermore, studies on human peripheral blood incubated for 16 h with 13 nm GNPs showed no increase in IL-1β in white blood cells (WBCs) at any of the concentrations used: 25, 50, 75, and 100 µg/mL [62].

Given the limited number of publications in the literature examining the effect of gold nanoparticles on blood leukocyte reactivity, this work represents a significant contribution to the current state of knowledge. The investigation of two shapes, three different nanoparticle concentrations, and two incubation times is a further strength of the current study.

To the best of our knowledge, the only other related articles in the literature describe studies on one nanoparticle type of different sizes [31], one size at three concentrations and two incubation times [32], one size at three concentrations [33], or three sizes at one concentration [35,36].

Toxicological studies of nanoparticles have shown that the physicochemical properties of nanoparticles, such as their size, shape, surface coating, surface charge, solubility, and chemical composition, can affect the behavior of the nanoparticles in biological systems and thus the toxicity of the nanoparticles [49,68,88]. The type of cells used in the study and the nanoparticle incubation time also affect the induced toxicity. Similarly, the dosage and method of administration of the nanoparticles also seem to be important [88].

Whole blood better reflects the physiological composition of cells found in the human body than isolated and purified cell subpopulations. It also creates an environment most similar to physiological conditions. However, it is necessary to take into account the presence of individual characteristics regarding the number of cells in the immune system of each individual person [89,90]. Therefore, slightly different results on cellular action modulation could be expected in in vivo studies. This justifies the partial inconsistency of the research results with the literature data, occurring mainly due to differences in the research model used, as most of the research work has been conducted on cell lines. The data from studies conducted in vitro do not provide complete results that can easily be extrapolated to the findings of in vivo studies regarding the effects of nanoparticles [88].

There are also some notable discrepancies regarding the method for synthesis, as well as the size and concentration of the nanoparticles used by researchers, further limiting the ability to compare our findings with those of others.

## 5. Conclusions

In summary, the GNPs used in our study did not significantly affect the production of reactive oxygen species by the granulocytes and monocytes without PMA stimulation, though they enhanced the stimulation of respiratory burst activity after the addition of PMA. Incubation with spherical gold nanoparticles resulted in enhanced induction of pyroptotic cells. Incubation with rod-like gold nanoparticles caused a slight increase in the induction of pyroptosis. There was little change in the IL-1β levels following 24 h incubation with the GNPs. Most of the relevant literature describes research carried out on cell lines, and ours is the only publication in which human peripheral blood was used as a research model. Therefore, this publication constitutes a significant extension of knowledge on this topic.

The use of different concentrations, shapes, and incubation times allowed us to explore different aspects of cell–NP interactions, and the results suggest that these nanoparticles could potentially be used for in vivo studies.

## Figures and Tables

**Table 1:** The characteristics of the blood types and Rh system of the study participants.

Sex	Number of Cases	Age (Mean ± Standard Deviation)	Blood Type				Rh Antigen	
			0	A	B	AB	+	−
Female	6	33 ± 5	3	1	2	0	5	1
Male	16	38 ± 7	5	5	6	0	14	2

## Data Availability

The data used to support the findings of this study is included in the article. The data will not be shared on the basis of third-party rights and commercial confidentiality.

## Supplementary Materials

The following supporting information can be downloaded at https://www.mdpi.com/article/10.3390/antiox13020157/s1. Figure S1: UV–Vis spectrum of spherical gold nanoparticles (A) (λ_max_ = 526 nm; optical path = 10 mm) and rod-shaped gold nanoparticles (B) (high peak λ = 685 nm, low peak λ = 518 nm; optical path = 10 mm). Figure S2: High-resolution transmission microscope image of spherical gold nanoparticles (A), with an average diameter of 13.24 ± 1.80 nm, and rod-shaped gold nanoparticles (B), with an average diameter of 31.92 ± 4.47 × 11.85 ± 3.13 nm. Figure S3: Thermogravimetric analysis results for spherical (GNP) and rod-like (GNR) gold nanoparticles. Figure S4: Reactive oxygen species production by granulocytes (A) and monocytes (B) incubated with spherical gold nanoparticles and by granulocytes (C) and monocytes (D) incubated with rod-shaped gold nanoparticles, evaluated based on the rhodamine 123 fluorescence intensity measured using flow cytometry. Figure S5: Reactive oxygen species production by granulocytes (A) and monocytes (B) incubated with spherical gold nanoparticles and by granulocytes (C) and monocytes (D) incubated with rod-shaped gold nanoparticles after stimulation with PMA, evaluated based on the rhodamine 123 fluorescence intensity measured using flow cytometry. Figure S6: Caspase-1 activity of granulocytes (A) and monocytes (B) incubated with spherical gold nanoparticles and of granulocytes (C) and monocytes (D) incubated with rod-shaped gold nanoparticles, evaluated based on the percentage of pyroptotic cells measured using flow cytometry. Figure S7: The concentration of interleukin-1β in the blood samples after incubation with spherical gold nanoparticles (A) and rod-shaped gold nanoparticles (B) for 24 h measured using an ELISA test.

## Appendix A. Description of the Synthesis of Spherical Gold Nanoparticles

The spherical nanoparticles were obtained using a one-step bottom-up in situ reaction, where the precursor was HAuCl_4_ and the reducer was sodium citrate [37]. Sodium citrate was used to reduce HAuCl_4_ in aqueous solution. It is not possible for sodium citrate to reduce HAuCl_4_ into metallic gold when the reaction solution is cold; this reaction takes place only once it has been heated. For this purpose, 17.623 mL of an aqueous solution of 1% HAuCl_4_ in 500 mL of Milli-Q water was used. The solution was placed on a heating plate and brought to the boil while stirring (with a heat plate temperature of approx. 170 °C). Next, 51.762 mL of solution containing 0.5966 g of sodium citrate was rapidly added with vigorous stirring. If successful, the solution should change in color from clear to yellow to dark red. After the addition of the sodium citrate, the solution was stirred and heated for 45 min. Upon cooling to room temperature, the solution was stirred once the heat had dissipated. The reaction mixture then adopted the color characteristic of the presence of spherical gold nanoparticles. After being cooled, the solution was filtered through syringe filters. The coating of the gold spherical nanoparticles with PEG-SH was performed according to previously described procedure [38]. The nanoparticles prepared this way were functionalized using PEG-SH (Mw ≈ 2000). This treatment prevents the formation of aggregates and improves the stability of the nanoparticles. For this purpose, a small amount of PEG-SH, previously sonicated for 30 min at 40 °C, was added to the nanoparticle solution. The solution was left on the stirrer for 24 h to allow the exchange of citrate ligands with the PEG-SH. Excess PEG-SH was then removed using centrifugation at 6000 rpm for about 30 min (twice) and it was washed to remove unreacted reagents. Centrifuged fractions of the PEGylated nanoparticles were transferred and suspended in saline solution.

## Appendix B. Description of the Synthesis of Rod-Like Gold Nanoparticles

Gold nanorods were obtained according to two-step bottom-up in situ reactions using the seed-mediated growth method proposed by Nikoobakth et al. [41], with some modifications [39,40]. Gold seeds (seed solution) were prepared in the first step. A transparent solution was obtained by dissolving CTAB (5 mL, 0.2 M) surfactant in deionized water and then adding the precursor, HAuCl_4_ (5 mL, 0.0005 M), and stirring for 10 min. Next, the cooled reducing agent, NaBH_4_ (0.6 mL, 0.01 M), was added and vigorously stirred, after which the mixture was allowed to stir for 1.5 h. All the reagents were added very quickly (one-shot). As NaBH_4_ must be freshly prepared and added before bubbling appears, we used water that had been frozen in a freezer ahead of time. The color of the mixture shifts from yellow to brownish–yellow. The seed solution was kept at 28 °C to avoid the crystallization of the CTAB surfactant. The second step in the synthesis is the growth of nanorods in solution, where 12 µL of the seed solution was added to the growth solution containing CTAB (5 mL, 0.2 M), HauCl_4_ (5 mL, 0.001 M), AgNO_3_ (0.075 mL and 0.25 mL, 0.004 M), and ascorbic acid (0.07 mL, 0.078 M). The seed solution should be added at a temperature of 27–30 °C to allow the growth of the gold nanorods. After adding the reducing agent to the CTAB, the solution was stirred until it became clear orange. The size and position of the LSPR gold nanorods can be controlled by changing the amount of AgNO_3_ used. Before further modification, the gold nanorods were centrifuged twice (6000 rpm for 30 min) to remove excess reactants. The PEG-SH coating was prepared using a modified version of a previously described method [91,92]. An aqueous solution of PEG-SH was sonicated for 30 min at 40 °C and added to the vigorously stirred solution of gold nanoparticles. The mixture was stirred for 24 h at room temperature to allow for the complete exchange of ligands with the PEG-SH. The minimal quantity of PEG-SH required to stabilize the surface of gold nanorods was calculated based on Manson et al. [93]. The unbound PEG-SH particles were then removed using centrifugation at 6000 rpm for 30 min, the supernatant was discarded, and the nanoparticles in the pellet were dispersed in Milli-Q water, transferred, and suspended in saline solution.
