# Supplementary material for: Effects of Spherical and Rod-like Gold Nanoparticles on the Reactivity of Human Peripheral Blood Leukocytes
Source: Antioxidants (Basel). 2024 Jan 26;13(2):157. doi: 10.3390/antiox13020157 (PMC10885998; doi:10.3390/antiox13020157)
Supplement: Supplementary file 1 [file antioxidants-13-00157-s001.zip › antioxidants-2826725-supplementary.pdf]

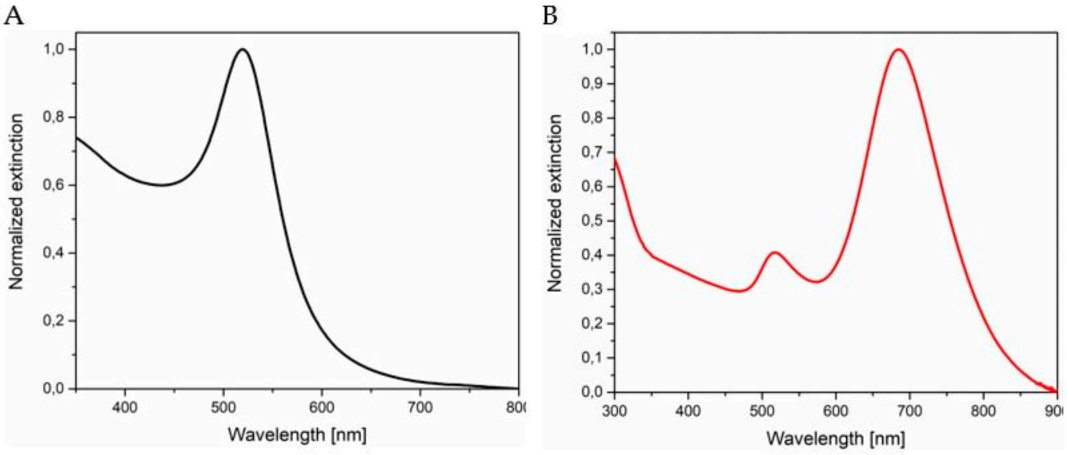

Figure S1. UV-Vis spectrum of spherical gold nanoparticles (A), λ_{max}=526 nm, optical path=10 mm and rod-shaped gold nanoparticles (B), high peak λ=685 nm, for low peak λ=518 nm, optical path=10 mm.

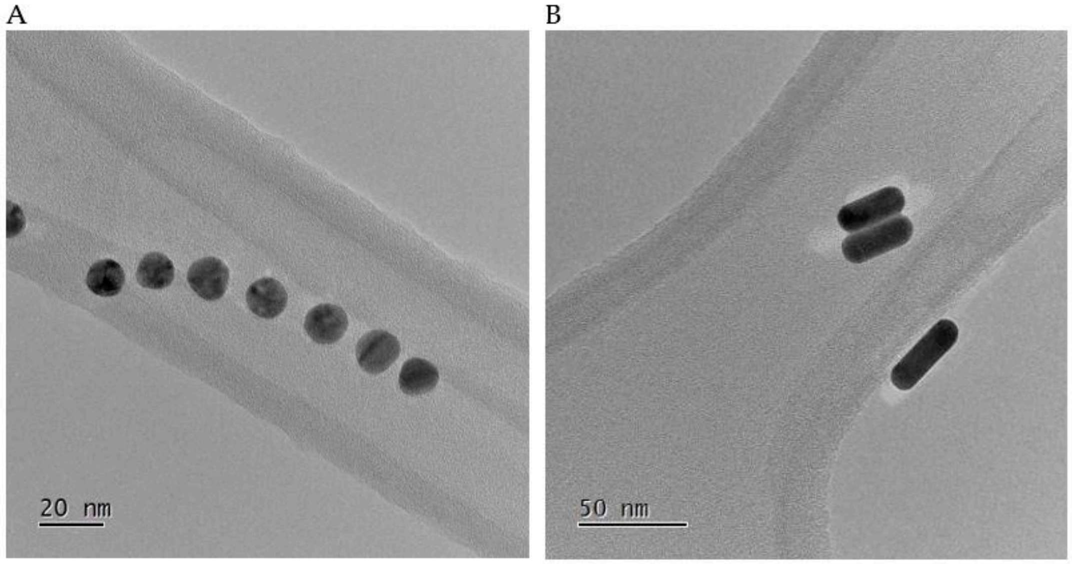

Figure S2. High-resolution transmission microscope image of spherical gold nanoparticles (A), with an average diameter of 13.24 ± 1.80 nm and rod-shaped gold nanoparticles (B), with an average diameter of 31.92 ± 4.47 x 11.85 ± 3.13 nm.

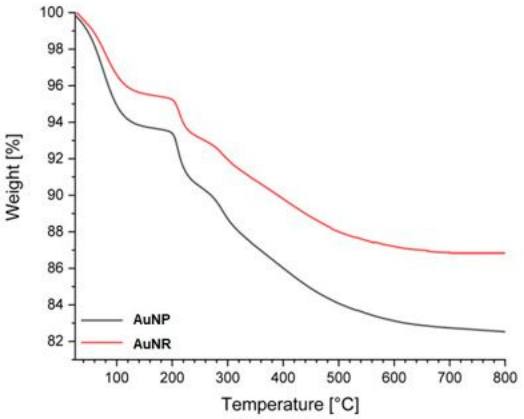

Figure S3. The thermogravimetric analysis result of spherical (GNP) and rod-like (GNR) gold nanoparticles.

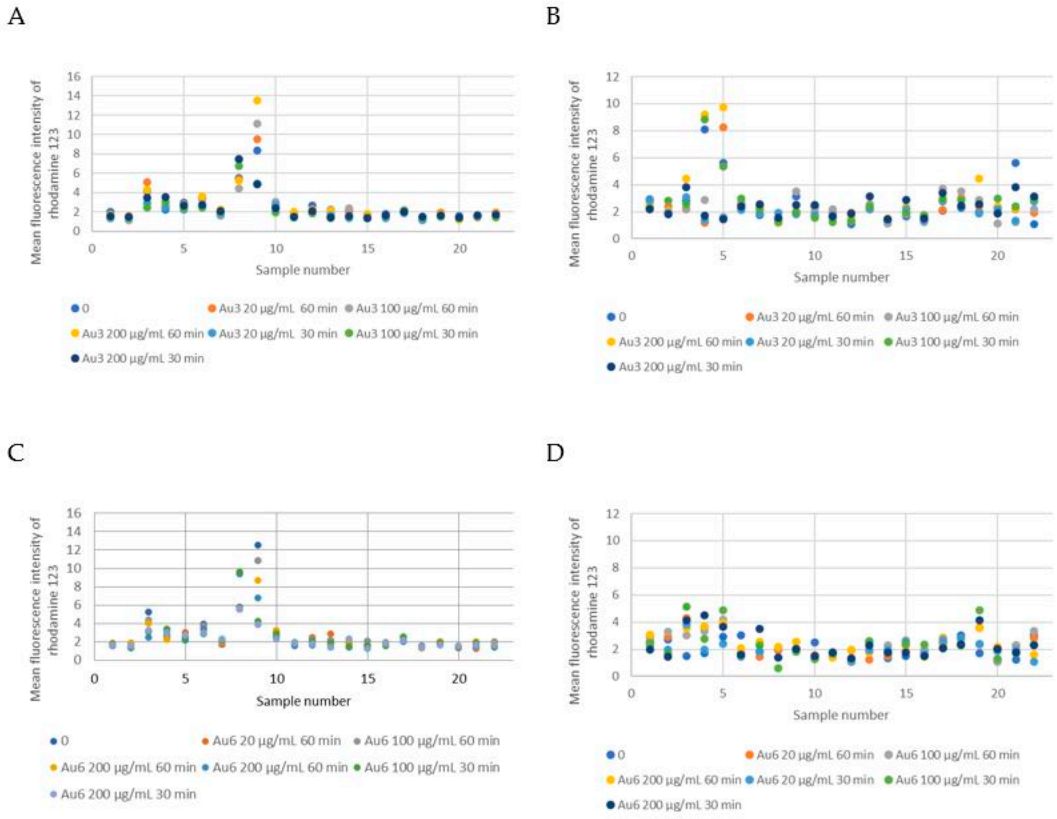

Figure S4. Reactive oxygen species production by granulocytes (A), monocytes (B) incubated with spherical gold nanoparticles and granulocytes (C), monocytes (D) incubated with rod-shaped gold nanoparticles, evaluated by the fluorescence intensity of rhodamine 123, measured by flow cytometry.

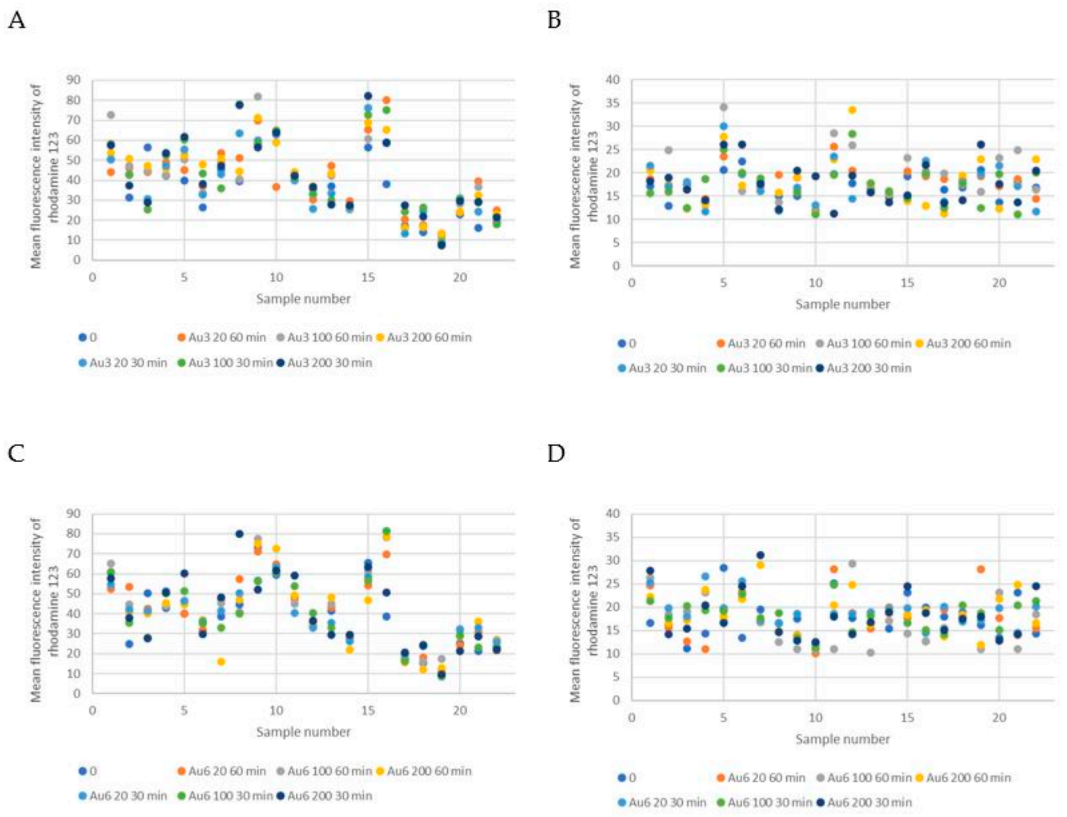

Figure S5. Reactive oxygen species production by granulocytes (A), monocytes (B) incubated with spherical gold nanoparticles and granulocytes (C), monocytes (D) incubated with rod-shaped gold nanoparticles, after stimulation with PMA, evaluated by the fluorescence intensity of rhodamine 123, measured by flow cytometry.

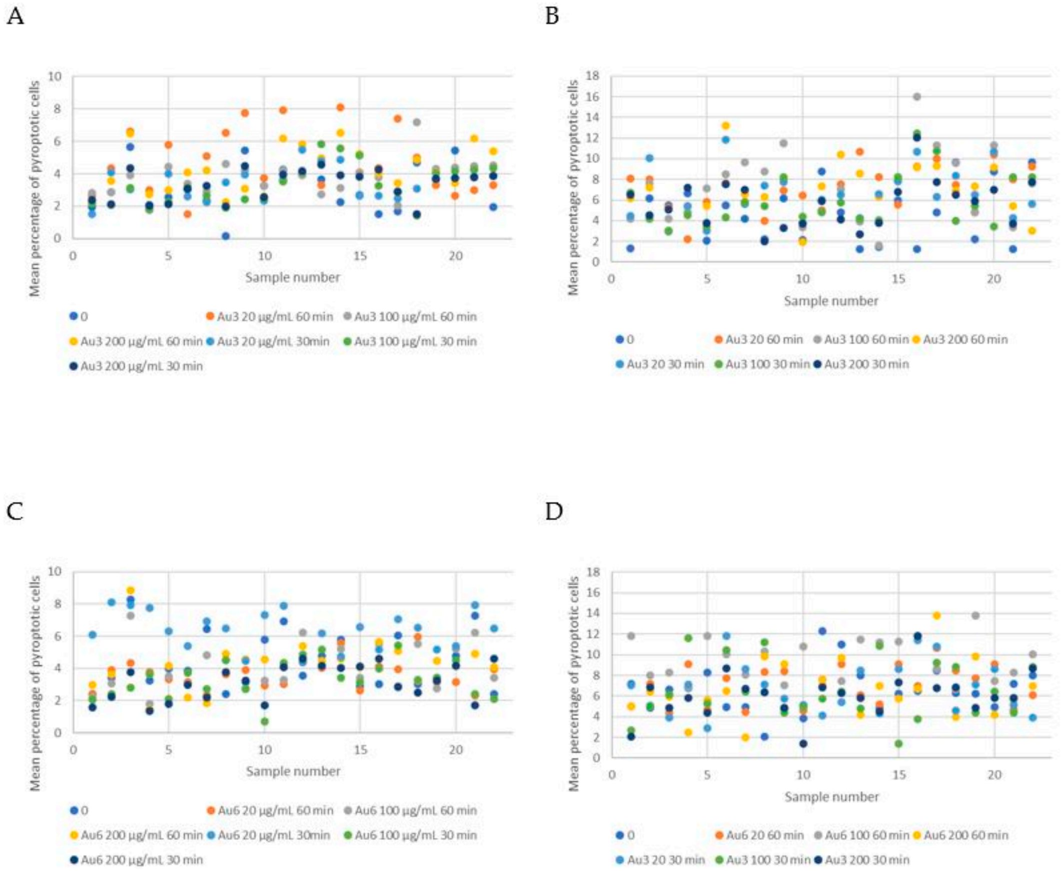

Figure S6. Caspase-1 activity of granulocytes (A), monocytes (B) incubated with spherical gold nanoparticles and granulocytes (C), monocytes (D) incubated with rod-shaped gold nanoparticles, evaluated by the percentage of pyroptotic cells, measured by flow cytometry.

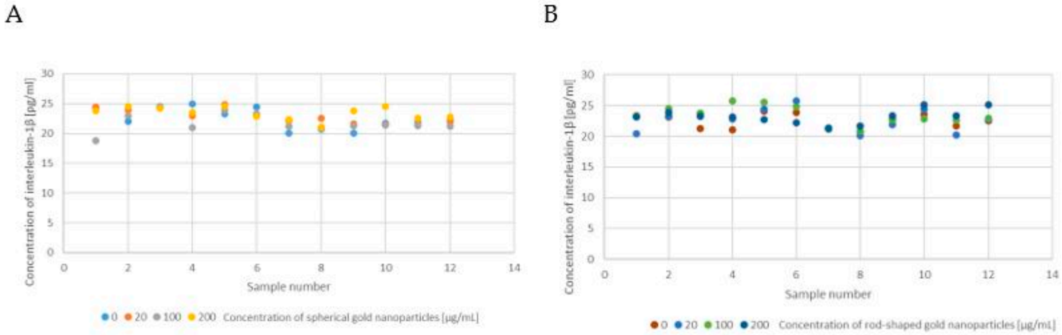

Figure S7. The concentration of interleukin-1β in blood samples after incubation with spherical gold nanoparticles (A) and rod-shaped gold nanoparticles (B) for 24 hours, measured by ELISA test.
